# Unveiling diabetic nephropathy: a novel diagnostic model through single-cell sequencing and co-expression analysis

**DOI:** 10.18632/aging.205982

**Published:** 2024-07-03

**Authors:** Guoyi Wang, Jinwen Zhao, Min Zhou, Haiyuan Lu, Fei Mao

**Affiliations:** 1 Department of Nephrology, The Affiliated Huaian No. 1 People's Hospital of Nanjing Medical University, Huai'an 223300, People's Republic of China; 2 Department of Urology, The Affiliated Huaian No. 1 People's Hospital of Nanjing Medical University, Huai'an 223300, People's Republic of China

**Keywords:** diabetic nephropathy, single-cell sequencing, transcriptome, diagnostic model, biomarkers

## Abstract

Background: Diabetic nephropathy (DN) is a severe complication of diabetes that affects the kidneys. Disulfidptosis, a newly defined type of programmed cell death, has emerged as a potential area of interest, yet its significance in DN remains unexplored.

Methods: This study utilized single-cell sequencing data GSE131882 from GEO database combined with bulk transcriptome sequencing data GSE30122, GSE30528 and GSE30529 to investigate disulfidptosis in DN. Single-cell sequencing analysis was performed on samples from DN patients and healthy controls, focusing on cell heterogeneity and communication. Weighted gene co-expression network analysis (WGCNA) and gene set enrichment analysis (GSEA) were employed to identify disulfidptosis-related gene sets and pathways. A diagnostic model was constructed using machine learning techniques based on identified genes, and immunocorrelation analysis was conducted to explore the relationship between key genes and immune cells. PCR validation was performed on blood samples from DN patients and healthy controls.

Results: The study revealed significant disulfidptosis heterogeneity and cell communication differences in DN. Specific targets related to disulfidptosis were identified, providing insights into the pathogenesis of DN. The diagnostic model demonstrated high accuracy in distinguishing DN from healthy samples across multiple datasets. Immunocorrelation analysis highlighted the complex interactions between immune cells and key disulfidptosis-related genes. PCR validation supported the differential expression of model genes VEGFA, MAGI2, THSD7A and ANKRD28 in DN.

Conclusion: This research advances our understanding of DN by highlighting the role of disulfidptosis and identifying potential biomarkers for early detection and personalized treatment.

## INTRODUCTION

Diabetic nephropathy, a severe complication of diabetes affecting the kidneys, has witnessed significant strides in recent years [1]. Scientists have delved into unraveling the intricate pathogenesis of the disease, investigating mechanisms involving inflammation, oxidative stress, and signaling pathways that contribute to kidney damage [2–4]. Clinical trials exploring various therapeutic interventions, including drugs targeting blood glucose levels and inflammation, have been conducted [5]. Renoprotective strategies, such as lifestyle modifications and pharmacological approaches, have been investigated to prevent or slow disease progression [6]. The emerging concept of precision medicine tailors treatments based on individual patient characteristics, and the integration of telemedicine and remote monitoring technologies aims to enhance patient care and early complication detection [7]. Stem cell therapy has also been explored for its regenerative potential in repairing damaged kidney tissue [8]. This multifaceted research landscape reflects a concerted effort to advance our understanding and management of diabetic nephropathy, with the ultimate goal of improving patient outcomes [9]. Currently, the key focus has been on identifying reliable biomarkers for early detection and monitoring, aiding in risk prediction and treatment assessment [10]. Genetic studies have advanced our understanding of the genetic factors influencing susceptibility to diabetic kidney disease, offering potential targets for personalized treatments.

Programmed cell death (PCD) is a crucial biological process that plays a fundamental role in the development, maintenance, and elimination of cells within multicellular organisms [11]. The intricate regulation of PCD is essential for various physiological functions, including tissue homeostasis, immune response, and embryonic development [12]. In recent years, significant strides have been made in unraveling the molecular mechanisms and signaling pathways involved in programmed cell death [13].

Disulfidptosis is a newly defined type of programmed cell death [14]. Researchers have found that in some diseases, SLC7A11 high expression cells are heavily consumed by NADPH, abnormal accumulation of cystine and other disulfides, which induces disulfide stress and rapid cell death [15]. This mechanism plays a critical role in maintaining cellular homeostasis and responding to stress conditions. In the context of disease, disulfidptosis has been implicated in the pathogenesis of various conditions, including neurodegenerative diseases, cancer, and cardiovascular disorders. The significance of disulfidptosis in disease lies in its potential as a therapeutic target. By modulating this pathway, it may be possible to control cell survival in pathological states, thereby offering a novel approach to treatment. For instance, in cancer, manipulating disulfidptosis could enhance the efficacy of chemotherapeutic agents by promoting cancer cell death. Similarly, in neurodegenerative diseases, protecting against excessive disulfidptosis may help preserve neuronal integrity and function. Understanding the regulatory mechanisms and molecular details of disulfidptosis is crucial for developing targeted therapies that can exploit this cell death pathway for clinical benefit. The ongoing research into disulfidptosis not only expands our knowledge of cell death mechanisms but also opens new avenues for the treatment of diseases where cell survival or death is dysregulated. However, the significance of disulfidptosis in diabetic nephropathy has been unclear [15].

Therefore, in this study, we combined single-cell sequencing data with bulk transcriptome sequencing data to explore the significance of disulfidptosis in diabetic nephropathy. For the first time, we reveal the disulfidptosis heterogeneity and cell communication landscape of diabetic nephropathy and identify specific targets. Our study can provide a reference for the early diagnosis and treatment of diabetic nephropathy.

## METHODS

### Single-cell sequencing data download and processing

The single-cell dataset GSE131882 associated with diabetic nephropathy was downloaded from the GEO database(https://www.ncbi.nlm.nih.gov/geo/) [16, 17]. The dataset included 3 diabetic nephropathy samples and 3 normal control samples. We used the “Seurat” package, version 4.3.0, to process and analyze single-cell data. The quality control of this study was as follows: (1) retention of genes larger than those expressed in 3 cells; (2) Cells that retain gene expression between 200 and 4000; (3) Cells that retain less than 15 percent of mitochondrial genes; (4) Cells with total gene expression less than 10000 were retained; The highly variable gene was set to 3000, and the “LogNormalize” method was used to standardize the data and integrate the sample. In this study, “PCA” analysis was first used to reduce the dimensionality of the data. “UMAP” then reduces the data dimension again and sets dim to 20. The KNN method was used to cluster the cells, setting dims to 20 and resolution to 0.4. We annotate the cells according to the signature genes of the cell type. The “UMAP” map was used to show the results of the single-cell analysis. We used the “AUCell” package to calculate the cell disulfidptosis fraction based on the concentration of the disulfidptosis gene set in the cell. The “Seurat” package’s “FindMarkers” function was used to analyze the differences between the two groups.

### Source of disulfidptosis-related gene set

Based on published articles, the disulfidptosis-related gene was summarized [14, 15]. These disulfidptosis-related genes are summarized in Supplementary Table 1.

### Bulk transcriptome data download and processing

Three diabetic nephropathy-related transcriptome datasets GSE30122, GSE30528 and GSE30529 were downloaded [18, 19]. The GSE30122 dataset contains 19 diabetic nephropathy samples and 50 normal control samples, and GSE30528 contains 9 diabetic nephropathy samples and 13 normal control samples. GSE30529 consisted of 10 diabetic nephropathy samples and 12 normal control samples. All data are standardized. The main analysis is performed in GSE30122, while the GSE30528 and GSE30529 datasets serve as validation sets for the later built models.

### Weighted gene co-expression network analysis (WGCNA)

We used the WGCNA method to explore the gene set closely associated with the disulfidptosis phenotype in diabetic nephropathy. The soft threshold ranges from 1 to 20. The pickSoftThreshold function looks for the appropriate soft threshold. The minimum number of module genes is set to 150, deepSplit = 2, and genes are clustered. We used the “ESTIMATE” package to calculate immune scores in diabetic nephropathy samples and correlated WGCNA-analyzed modules with disulfidptosis and immunophenotypes.

### Gene set enrichment analysis (GSEA)

Firstly, the differentially expressed genes of the diabetic nephropathy group and healthy controls were obtained. The results of the difference analysis were sorted according to the magnitude of the difference multiple, and then compared with some gene sets of specific functions to find the most closely related pathways.

### Enrichment analysis

Gene Ontology (GO) is a database established by the Gene Ontology Consortium, which aims to establish a database applicable to various species, to define and describe the function of genes and proteins, which is applicable to various species. There are three categories: Biological Process (BP), Cellular Component (CC) and Molecular Function (MF). Kyoto Encyclopedia of Genes and Genomes (KEGG) database is a database that systematically analyzes gene functions, linked genomic information and functional information, including metabolic pathway database, hierarchical classification database, gene database, genome database, etc. By comparing the studied genes with the pathway gene set in the database, the enriched pathways are obtained.

### The construction of the diagnostic model and nomogram

In this study, a variety of machine learning methods were used to construct a diagnostic model based on the “mlr3” package. Firstly, the features of included genes were screened by the “auc” method, the first 4 genes were retained, the external cyclic ressampling “CV” was set to 3, and the model results were compared to select the optimal model.

### Immunocorrelation analysis

CIBERSORT is a commonly used method to calculate transcriptome immune infiltration. In this study, the expression matrix of the dataset was compared with the built-in matrix of the method to obtain the degree of immune infiltration of each patient in the dataset, and then the relationship between four key genes and immune cells in diabetic nephropathy was explored.

### PCR experimental verification

Blood samples from 6 DN patients and 6 healthy controls were collected for PCR analysis. TRIzol reagents (Invitrogen, CA, USA) were used to extract total RNA, following the instructions provided by the manufacturer. This was followed by cDNA synthesis using the PrimeScript RT Reagent Kit (Takara, Nanjing, China). Finally, AceQ Universal SYBR qPCR Master Mix (Vazyme, Nanjing, China) was utilized to conduct quantitative real-time PCR (qRT-PCR). The primer sequence we used is shown in Supplementary Table 2.

### Statistical analysis

The Seurat R package (version 4.3.0) was used for single-cell sequencing analysis. AUCell R packages are used to calculate gene set activation in different cells. The WGCNA R package is used for WGCNA. The limma package was used for differential expression analysis. The mlr3 R package is used to build the diagnostic model. The expression of differential genes between the two groups was measured by the rank-sum test, and the correlation between genes and immune cells was measured by Pearson’s method. *p* < 0.05 was defined as statistically significant.

## RESULTS

### Single-cell sequencing analysis

**Figure 1:**
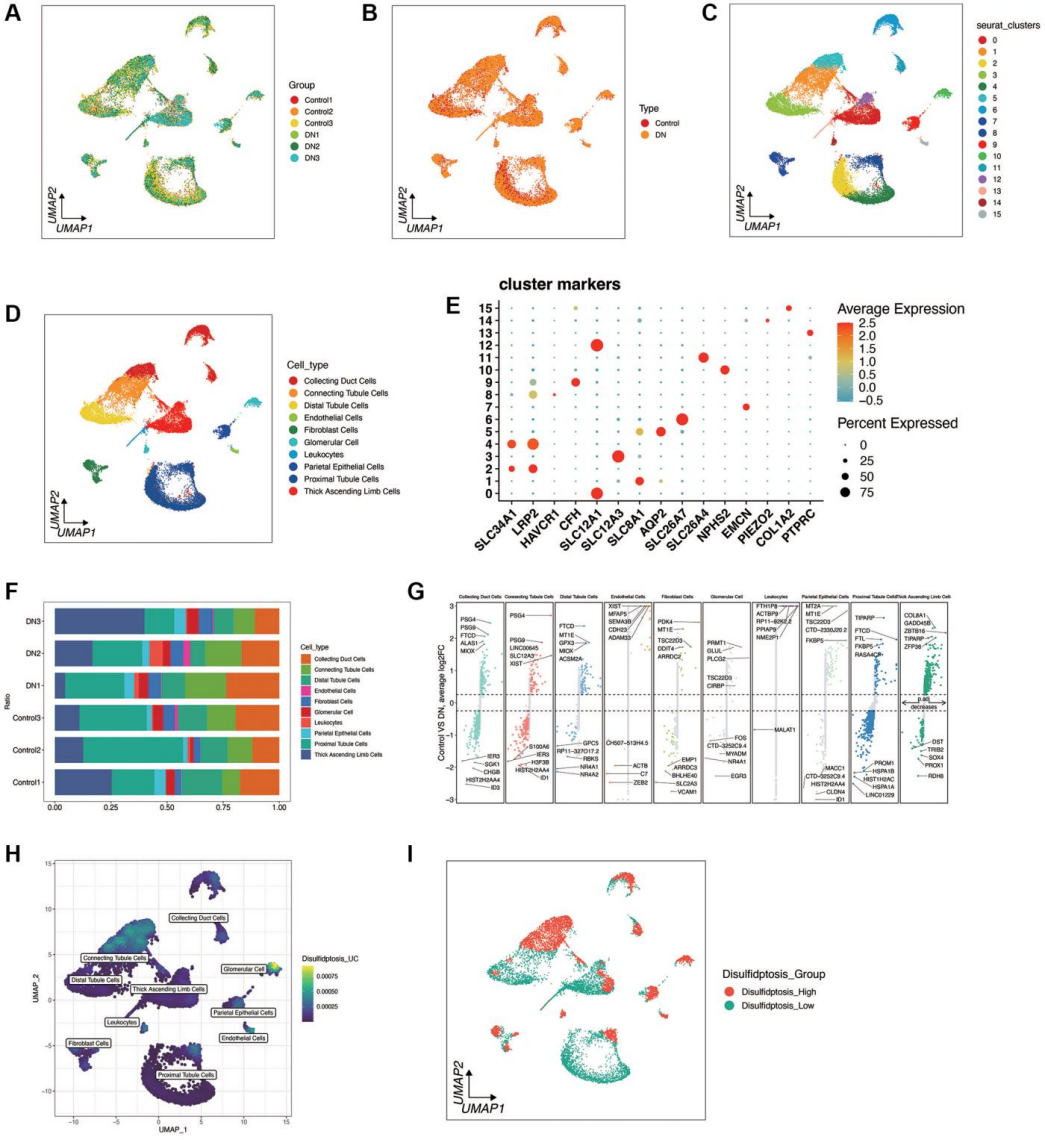
**Single-cell sequencing analysis of diabetic nephropathy (DN) and control samples.** (**A**, **B**) Quality control metrics and sample integration results showing no significant batch effects between DN and control samples, with observed heterogeneity. (**C**–**E**) UMAP plots illustrating cell clustering into 16 distinct clusters based on cell type marker genes, with 10 cell types annotated. (**F**, **G**) Distribution of cell types across samples and the top 5 significantly up-regulated and down-regulated differential genes for each cell type in DN and control groups. (**H**, **I**) Enrichment fraction of disulfidptosis in Glomerular Cells and Connecting Tubule cells, with samples categorized into Disulifidptosis_High and Disulifidptosis_Low groups based on median values, highlighting differentially expressed genes between the two groups.

Disulfidptosis was investigated at the single-cell level of diabetic nephropathy. As shown in Figure 1A, 1B, a total of 3 diabetic nephropathy and 3 control samples were retained through quality control and sample integration. There was no obvious batch effect between the samples of diabetic nephropathy and the samples of the control group, and heterogeneity was observed between the diabetic nephropathy and the control samples. As shown in Figure 1C–1E, cells were clustered into 16 clusters according to the marker genes of cell types, and a total of 10 cell types were annotated. Figure 1F, 1G shows the distribution of cell types in each sample, as well as the top 5 most significant up-regulated and down-regulated differential genes of each cell type in both the diabetic nephropathy and the control group. As shown in Figure 1H, 1I, the death enrichment fraction of double flow was mainly in glomerular cells and connecting tubule cells, and was divided into disulfidptosis high group and disulfidptosis low group according to the median value. Different genes between the two groups were found.

### Functional enrichment analysis

**Figure 2:**
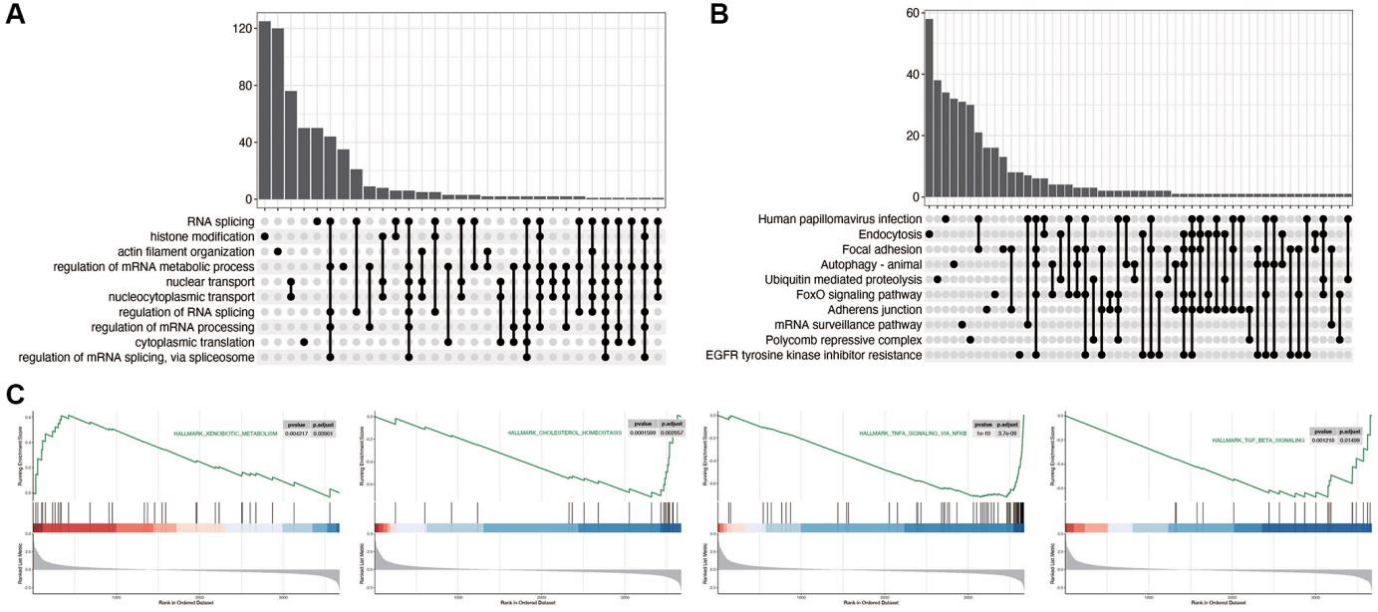
**Functional enrichment analysis in diabetic nephropathy.** (**A**, **B**) Pathway analysis comparing DN to control group, indicating significant enrichment in RNA splicing, histone modification, Focal adhesion, and FoxO signaling pathways in DN. (**C**) Pathways related to disulfidptosis in DN, showcasing activation of the XENOBIOTIC_METABOLISM pathway and inhibition of other key pathways.

As shown in Figure 2A, 2B, we first explored the relevant functional pathways in diabetic nephropathy compared with the control group, and found that RNA splicing, histone modification, Focal adhesion and FoxO signaling pathways were mainly enriched in diabetic nephropathy. We also subsequently explored the pathways related to disulfidptosis in diabetic nephropathy. As shown in Figure 2C, the xenobiotic metabolism pathway is activated, while the other three pathways are inhibited.

### WGCNA analysis

**Figure 3:**
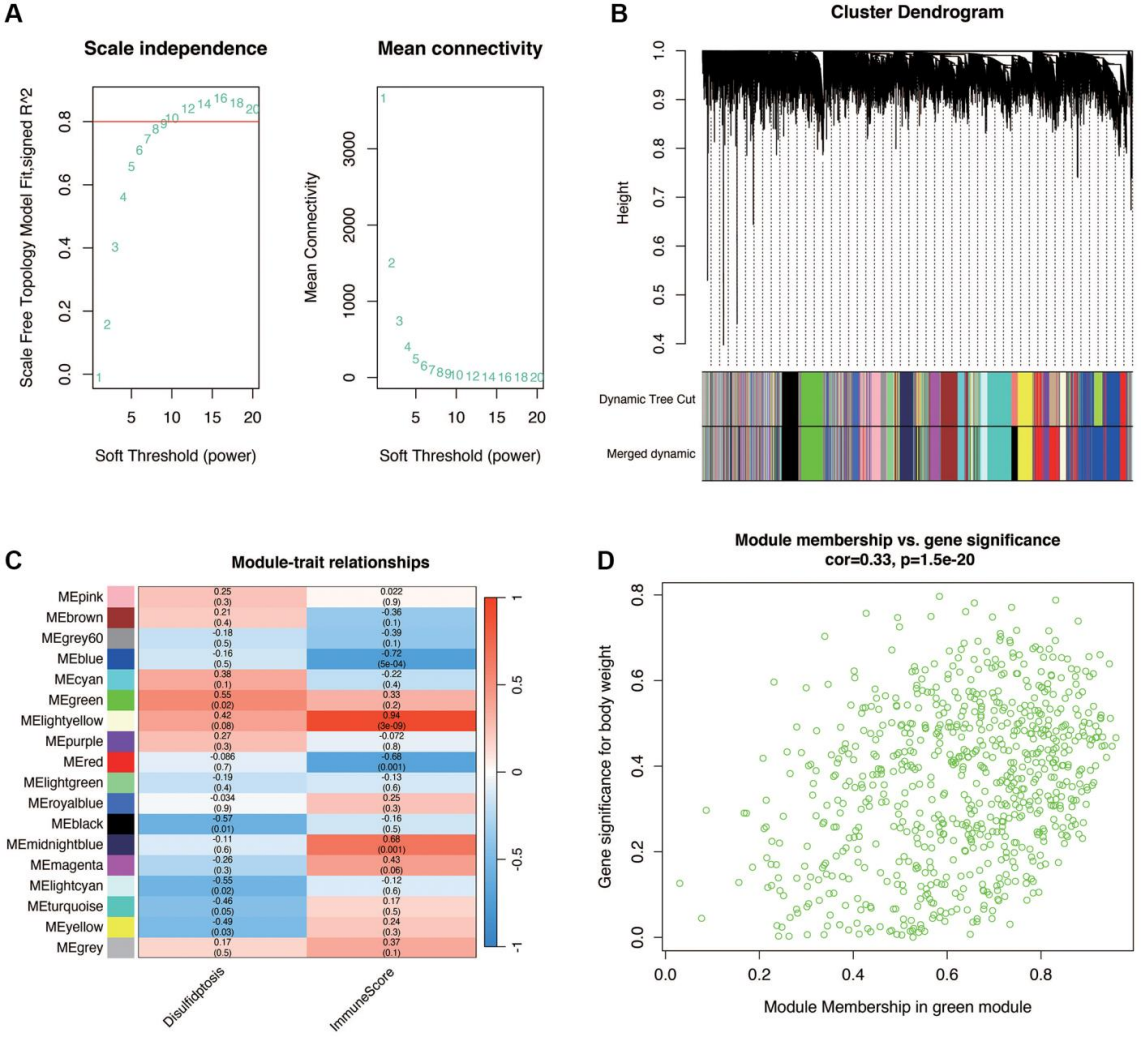
**Weighted gene co-expression network analysis (WGCNA) in DN.** (**A**) Selection of the optimal soft threshold (power of 14) ensuring data conforms to the power law distribution for network construction. (**B**, **C**) Gene clustering into 17 non-gray modules, with the green module showing the highest association with disulfidptosis phenotype (correlation and statistical significance). (**D**) Positive correlation between gene significance for disulfidptosis and module membership within the green module, indicating relevance to DN pathogenesis.

In diabetic nephropathy, to further obtain genes associated with disulfidptosis, WGCNA analysis was performed in the GSE30122 dataset. As shown in Figure 3A, when the optimal soft threshold is 14, the data conforms to the power law distribution. As shown in Figure 3B, 3C, the genes were clustered into 17 non-gray modules, with the green module most associated with the disulfidptosis phenotype (cor = 0.55 and *p* < 0.05). We further explored the inter-gene correlation in the green module, as shown in Figure 3D. There was a positive correlation between Module membership in green module and Gene significance for body weight (cor = 0.33 and *p* < 0.05).

### The key disulfidptosis gene in diabetic nephropathy was obtained

**Figure 4:**
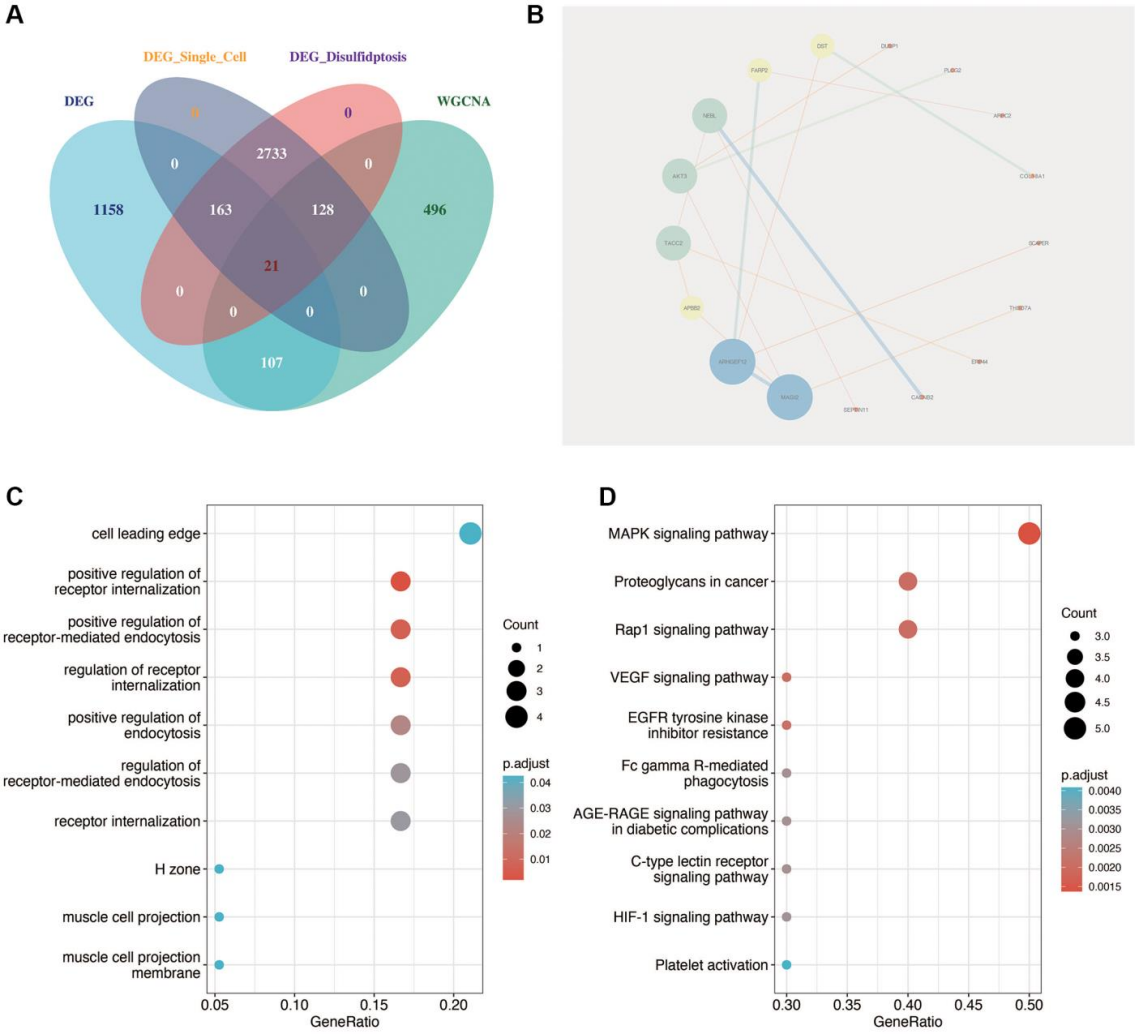
**Identification of key disulfidptosis-related genes in DN.** (**A**) Intersection analysis identifying 21 critical genes related to disulfidptosis in DN through single-cell analysis, differential expression, and WGCNA. (**B**) Protein interaction network analysis highlighting MAGI2 as a central factor in gene-gene interactions. (**C**, **D**) Functional enrichment analysis of the 21 key genes, showing significant association with receptor internalization and MAPK signaling pathway.

As shown in Figure 4A, the key genes of diabetic nephropathy obtained by single-cell analysis were intersects with the differentially expressed genes of GSE30122 and the genes related to disulfidptosis of diabetic nephropathy obtained by WGCNA analysis, and a total of 21 relatively important genes were obtained. Then, we carried out protein interaction analysis using the “STRING” website and visualization using cytoscape software. As shown in Figure 4B, MAGI2 was the most important factor in the correlation between genes. As shown in Figure 4C, 4D, the functional enrichment analysis of these 21 genes showed that they were mainly related to positive regulation of receptor internalization and MAPK signaling pathway.

### Construction of a diagnostic model

**Figure 5:**
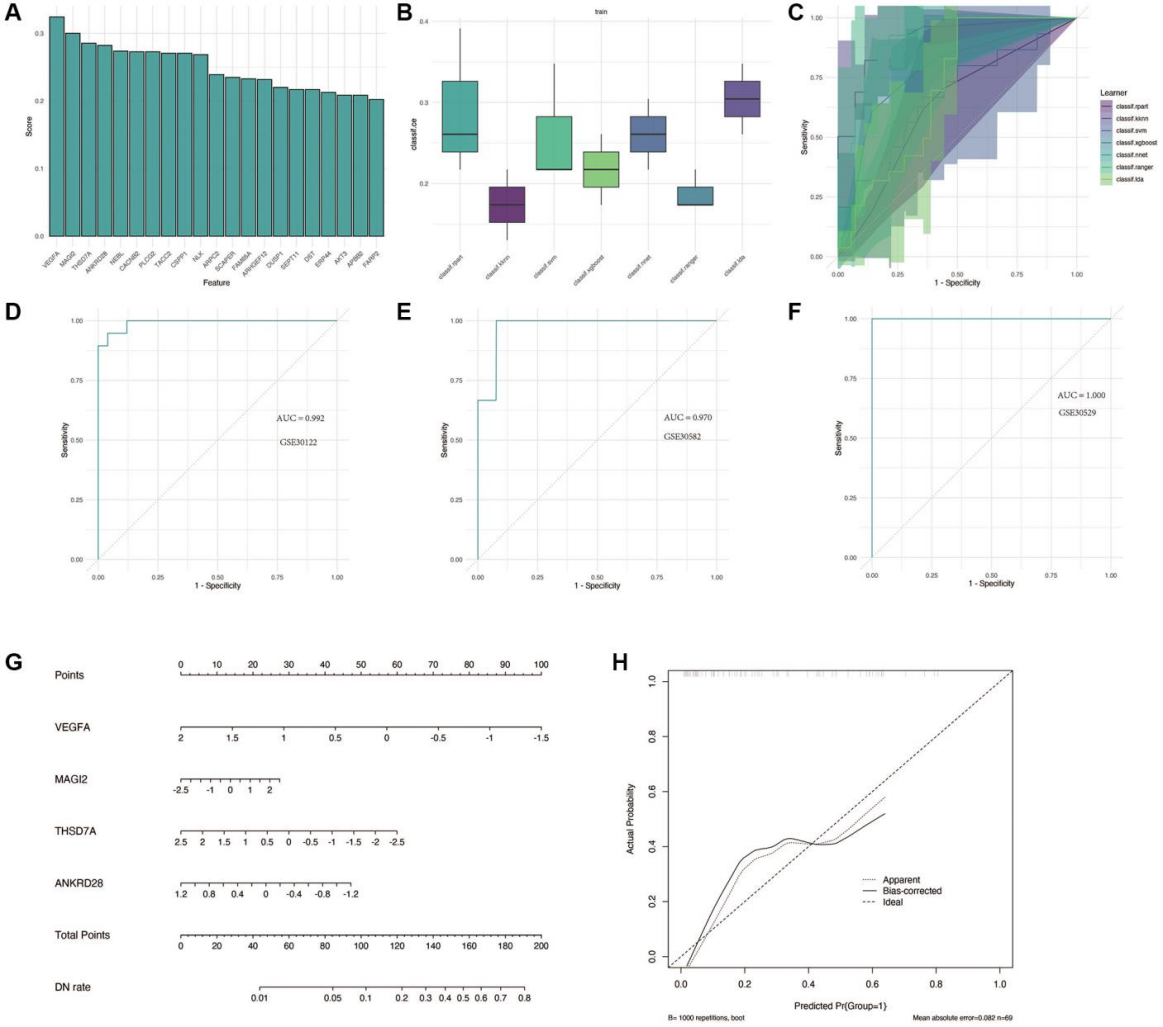
**Construction and validation of the diagnostic model for DN.** (**A**) Identification of the four most significant genes (VEGFA, MAGI2, THSD7A, and ANKRD28) through feature screening. (**B**, **C**) Comparison of machine learning models, with the KNN method showing superior accuracy and AUC in distinguishing DN from control samples. (**D**–**F**) Performance evaluation of the diagnostic model in training and validation cohorts (GSE30122, GSE30528, and GSE30529) with AUC >0.9. (**G**, **H**) Nomogram construction for clinical application, enabling accurate prediction of DN risk based on gene expression.

We performed the analysis in GSE30122. As shown in Figure 5A, the first four important genes were VEGFA, MAGI2, THSD7A and ANKRD28. Subsequently, a variety of machine learning methods were used and compared, as shown in Figure 5B, 5C. Compared with other models, “KNN” method has the highest accuracy and the largest AUC area. As shown in Figure 5D–5F, this model performs well in training cohort GSE30122 and verification cohorts GSE30528 and GSE30529, with AUC greater than 0.9. For better clinical application, we constructed a nomogram, as shown in Figure 5G, 5H, to accurately predict the possibility of diabetic nephropathy by combining the expression of four genes.

### Immunocorrelation analysis

**Figure 6:**
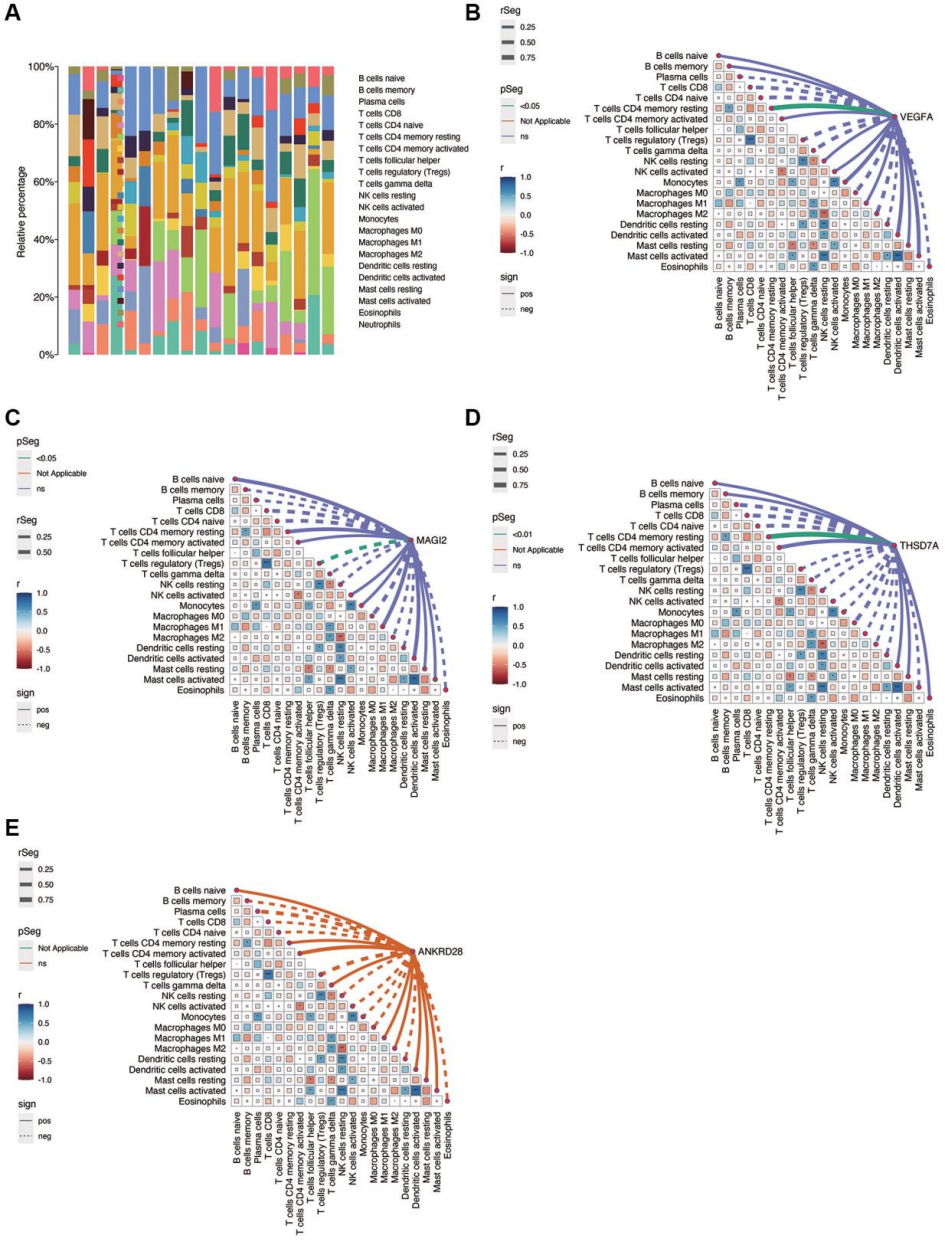
**Immunocorrelation analysis in DN.** (**A**) Overview of immune cell infiltration in DN samples. (**B**–**E**) Correlation analysis between immune cell populations and the expression of key genes (VEGFA, MAGI2, THSD7A), revealing significant associations and suggesting potential immunomodulatory roles in DN.

Figure 6A shows the immune infiltration of each sample in diabetic nephropathy. As shown in Figure 6B–6E, there is a correlation between immune cells, in which NK Cell resting is positively correlated with Macrophage M2, while Tregs are negatively correlated with T cells CD8. NK Cell resting was negatively correlated with Mast cells activated and Dendritic cells resting. VEGFA was positively correlated with T cells CD4 memory resting (*p* < 0.05), MAGI2 was negatively correlated with Tregs (*p* < 0.05), and THSD7A was positively correlated with T cells CD4 memory resting (*p* < 0.05). No significant correlation was found between ANKRD28 and immune cells.

### Expression analysis of model genes and PCR validation

**Figure 7:**
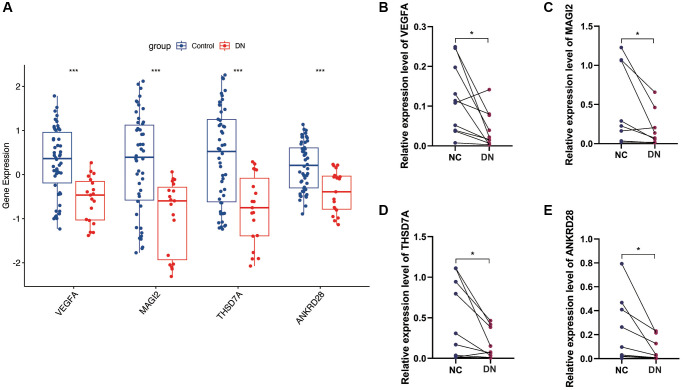
**Gene expression analysis and PCR validation.** (**A**) Expression analysis of the four model genes in the GSE30122 cohort, showing down-regulation in DN samples. (**B**–**E**) PCR validation results confirming the reduced expression levels of VEGFA, MAGI2, THSD7A, and ANKRD28 in DN compared to control blood samples, supporting their potential as diagnostic biomarkers. (^*^p < 0.05, ^***^p < 0.001).

In the GSE30122 cohort, we analyzed the expression of four model genes, VEGFA, MAGI2, THSD7A and ANKRD28, and found that the expression of these model genes was down-regulated in the DN group (Figure 7A). Subsequently, we conducted PCR validation in clinical samples and also found down-regulated expression levels of VEGFA, MAGI2, THSD7A and ANKRD28 in the DN group (Figure 7B–7E) (^*^p < 0.05, ^***^p < 0.001).

## DISCUSSION

The intricate landscape of diabetic nephropathy (DN), a notable complication of diabetes mellitus, underscores a compelling need for advancing our understanding of its pathophysiology and identifying novel diagnostic and therapeutic targets [20–22]. The current study illuminates the role of disulfidptosis, a recently characterized form of programmed cell death, within the context of DN, offering fresh insights into the cellular mechanisms underpinning kidney damage in diabetes. By integrating single-cell sequencing with bulk transcriptome data, we have elucidated the heterogeneity of disulfidptosis and its cellular communication landscape in DN, thereby identifying potential biomarkers and therapeutic targets.

The exploration of DN pathogenesis has traditionally focused on mechanisms such as inflammation, oxidative stress, and aberrant signaling pathways. Our study expands this understanding by spotlighting disulfidptosis, a form of cell death induced by disulfide stress, which has been relatively unexplored in DN. The findings reveal that disulfidptosis contributes significantly to the cellular heterogeneity observed in DN, suggesting that its modulation could offer new avenues for treatment.

Single-cell sequencing analysis offered a granular view of the cellular composition in DN, unveiling distinct patterns of gene expression and cell-type-specific differences between diseased and healthy kidney tissue. This high-resolution mapping underscores the complex interplay between various cell types in the kidney, each contributing uniquely to the pathogenesis of DN. The identification of disulfidptosis predominantly in glomerular and connecting tubule cells highlights specific cellular contexts where disulfidptosis might play a pivotal role, warranting further investigation into its mechanistic underpinnings and potential as a therapeutic target.

Our use of weighted gene co-expression network analysis (WGCNA) to correlate gene expression modules with disulfidptosis phenotype provides a robust framework for identifying genes and pathways associated with this form of cell death in DN. The association of specific gene modules with disulfidptosis not only confirms its relevance in DN but also identifies potential molecular signatures that could serve as biomarkers for early detection or targets for therapeutic intervention.

The functional enrichment analysis further delineates the biological pathways associated with disulfidptosis in DN, such as RNA splicing, histone modification, and the MAPK signaling pathway. These findings are consistent with the known complexity of DN pathogenesis and suggest that disulfidptosis might intersect with other cellular processes contributing to kidney damage. Understanding these pathways in detail could lead to the development of multi-targeted therapeutic strategies that address various aspects of DN pathophysiology.

The construction of a diagnostic model based on key disulfidptosis-related genes represents a significant leap towards the clinical translation of our findings. The model’s high accuracy in differentiating DN from control samples across multiple datasets underscores its potential utility in clinical settings. Moreover, the development of a nomogram for predicting DN risk based on gene expression profiles exemplifies the practical application of our research, offering a tool that could enhance early detection and personalized management of DN.

Immunocorrelation analysis in our study sheds light on the complex interactions between the immune system and kidney cells in the context of DN. The correlation between key genes and specific immune cell populations suggests that disulfidptosis might also influence the immune landscape in DN, an aspect that warrants further exploration. Understanding how disulfidptosis affects immune cell behavior could provide insights into the inflammatory processes in DN and reveal new targets for modulating the immune response to slow disease progression.

Our PCR validation of model genes in clinical samples reinforces the relevance of these genes in DN, supporting their potential as biomarkers for disease detection and monitoring. The downregulation of genes such as VEGFA, MAGI2, THSD7A, and ANKRD28 in DN samples aligns with the notion that disulfidptosis and its associated molecular pathways are intricately involved in the pathogenesis of DN.

This study is not without limitations. While it provides a comprehensive analysis of disulfidptosis in DN, the mechanisms by which disulfidptosis contributes to kidney damage remain to be fully elucidated. Future studies should aim to unravel the molecular events leading to disulfidptosis in kidney cells and explore the therapeutic potential of modulating this process. Additionally, the translational impact of our findings would benefit from validation in larger cohorts and through experimental models that can mimic the complexity of DN.

## CONCLUSIONS

In conclusion, our study represents a significant advance in the understanding of DN pathogenesis, highlighting the role of disulfidptosis in the disease process. By integrating cutting-edge genomic analysis techniques, we have identified potential biomarkers and therapeutic targets that pave the way for novel diagnostic and treatment strategies. The findings underscore the importance of exploring new forms of programmed cell death in chronic diseases and open up new avenues for research into the cellular mechanisms of DN. As we continue to unravel the complexities of DN, the insights gained from this study will undoubtedly contribute to the development of more effective approaches to manage this challenging condition, ultimately improving patient outcomes.

## Supplementary Materials

Supplementary Tables
