# Supplementary material for: Unveiling diabetic nephropathy: a novel diagnostic model through single-cell sequencing and co-expression analysis
Source: Aging (Albany NY). 2024 Jul 3;16(13):10972–84. doi: 10.18632/aging.205982 (PMC11272118; doi:10.18632/aging.205982)
Supplement: Supplementary Tables [file aging-16-205982-s001.pdf]

SUPPLEMENTARY TABLES

Supplementary Table 1. Disulfidptosis-related genes.

Disulfidptosis
FLNA
FLNB
MYH9
TLN1
ACTB
MYL6
MYH10
CAPZB
DSTN
IQGAP1
ACTN4
PDLIM1
CD2AP
INF2
SLC7A11
SLC3A2
RPN1
NCKAP1
NUBPL
NDUFA11
LRPPRC
OXSM
NDUFS1
GYS1

Supplementary Table 2. PCR primer sequence.

	The primer sequence	
VEGFA	Forward	ACAACAAATGTGAATGCAGACCA
	Reverse	GAGGCTCCAGGGCATTAGAC
MAGI2	Forward	CCAGGTTTCCGAGAAAAACCA
	Reverse	CTCATCAGGCTCGTCTCCAC
THSD7A	Forward	GGAGTGGTGTGAAGGTTCGT
	Reverse	CCTCATACACCTGTGCCTGG
ANKRD28	Forward	TCACAGAACTGGCATGAACCT
	Reverse	GCACCAGTGATGGCAGAGAT
